# Compendium of the Ligaments; Illustrated by Woodcuts, with the Articular Cartilages, Interarticular or Moveable Fibro-Cartilages, Synovial Membranes, and Bursæ Mucosæ of the Joints, &c.

**Published:** 1836-01

**Authors:** 


Art. XI.—*Compendium of the Ligaments; illustrated by Woodcuts, with the Articular Cartilages, Interarticular or Moveable Fibro-Cartilages, Synovial Membranes, and Bursæ Mucosæ of the Joints; the Mode of Union, and the Bones entering into the Composition of each; and an Outline of the Dislocations, Fractures, Physiology, and Pathology.* By A. M'Nab, jun. m.r.c.s. l. 1835. pp. 86.

The little compilation bearing the above title, is a concise and tolerably well digested statement of the subjects of which it professes to treat. It can hardly be expected that in eighty-six pages of a small duodecimo anything beyond an outline of such subjects can be afforded; and it must require more than ordinary powers of judicious compression to render such an outline of much practical utility; the imperfections, therefore, of the present treatise are rather those of manuals in general, than of this particular performance. These objections are not applicable to the anatomical or physiological parts of the work; the former is well arranged and carefully executed; and the latter sufficient for all practical purposes. Of the woodcuts, we cannot speak favourably.

In that portion of his Compendium in which the author has treated of the anormal conditions of the parts, to the consideration of which his work is devoted, his object has evidently been to collect a great quantity of matter; and in so doing he has evinced considerable industry. The chief defects (in treating of dislocations and fractures,) consist in occasional omissions of the causes of such injuries, circumstances the knowledge of which assists so materially in diagnosis; in an insufficient notice of difficulties arising from complications; and in a deficiency on the subject of differential diagnosis. Of the diseases of bones and joints, a succinct and faithful account, as far as so much condensation will allow, has been given; but the remedial means are prescribed without sufficiently associating them with the conditions in which they should be individually employed. The author's style would admit of considerable improvement; the want of a proper employment of periods, and an indulgence in long sentences, giving a heaviness and confusedness to his composition.

We may recommend the work to those who require an accurate account of ligaments; but in its surgical department it can scarcely be of much utility.

